# Recombinant Alphaherpesvirus Vectors in Veterinary Vaccinology: Platforms, Applications, and Translational Challenges

**DOI:** 10.3390/ijms27135686

**Published:** 2026-06-24

**Authors:** Ali Mazloum, Sofya G. Feoktistova, Veronika Ledyaeva, Gava Khulkhachiev, Olga N. Mityaeva, Pavel Yu Volchkov

**Affiliations:** 1 Moscow Center for Advanced Studies, Kulakova Street 20, Moscow 123592, Russia; 2 Federal Research Center for Innovator and Emerging Biomedical and Pharmaceutical Technologies, Moscow 125315, Russia; 3 Moscow Clinical Scientific Center N.A. A.S. Loginov, Novogireevskaya Street, Moscow 111123, Russia; 4 Faculty of Fundamental Medicine, Lomonosov Moscow State University, Moscow 119991, Russia

**Keywords:** animal vaccinology, bovine herpesvirus type 1 (BoHV-1), duck enteritis virus (DEV), equine herpesvirus type 1 (EHV-1), Marek’s disease virus, One Health, pseudorabies virus (PRV), recombinant vaccine, vaccine platforms, viral vectored vaccines

## Abstract

Animal infectious diseases impose severe economic burdens on livestock industries, threaten wildlife populations, and compromise food security. Although vaccination remains the cornerstone of disease prevention, conventional vaccine platforms are often constrained by safety, efficacy, or manufacturing scalability. This narrative review provides a comprehensive analysis of the state of the art in herpesvirus-vectored vaccines for veterinary applications, focusing on five well-characterized alphaherpesviruses: Bovine herpesvirus type 1 (BoHV-1), Pseudorabies virus (PRV), Marek’s disease virus (MDV), Equine herpesvirus type 1 (EHV-1), and Duck enteritis virus (DEV). The intrinsic characteristics of herpesviruses, including large, stable genomes; the capacity for foreign gene insertion; broad host tropism; and the ability to elicit robust humoral and cellular immunity, are examined, and their performance is compared with that of traditional vaccine platforms. Key advances in vectored vaccine development are highlighted, from proof-of-concept studies to the creation of advanced multivalent constructs. These approaches demonstrate protective efficacy against a range of significant animal pathogens, including foot-and-mouth disease virus, porcine reproductive and respiratory syndrome virus, avian influenza virus, infectious bursal disease virus, and West Nile virus. The literature was identified through systematic searches of PubMed, Google Scholar, and Web of Science (1990–2026), followed by title/abstract screening and reference chaining. Future directions in vector engineering, mucosal delivery, and synthetic biology approaches are considered. Herpesvirus-vectored vaccines represent a versatile platform for enhancing animal health, supporting sustainable agriculture, and mitigating zoonotic risks.

## 1. Introduction

The economic impact of infectious animal diseases is profound, with outbreaks threatening the sustainability of livestock industries, endangering wild animal populations, undermining farmer livelihoods, and disrupting international trade [1]. In response, governments implement stringent biosecurity measures, including disease surveillance, trade restrictions, and strategic vaccination programs, to safeguard animal health, economic stability, and food security. Among these interventions, vaccination remains the most effective and proactive resource for disease prevention. It is thus of the utmost importance that a vaccine can elicit a robust and durable immune response.

The concept of vaccination has evolved significantly since the seminal work of Edward Jenner in 1798, who used cowpox to protect against smallpox, laying the foundation for modern vaccinology [2]. Today, a diverse arsenal of vaccine platforms exists, each with its intrinsic advantages and limitations. These include live-attenuated, inactivated, subunit, virus-like particle (VLP), and nucleic acid-based (DNA and mRNA) vaccines.

Inactivated vaccines often induce strong antibody responses but may require adjuvants/boosters and are generally weaker for cellular immunity. They also risk altering antigenic epitopes during inactivation, potentially compromising efficacy [3,4]. Live-attenuated vaccines often provide superior, long-lasting immunity by mimicking natural infection but carry inherent risks, including potential recombination or reassortment with virulent viruses, residual virulence, or reversion to a pathogenic phenotype, particularly with RNA viruses, which are prone to high mutation rates [5,6]. Subunit and VLP vaccines are notably safe and well-tolerated but often exhibit weaker immunogenicity, necessitating multiple boosters and potent adjuvants, which complicates manufacturing and deployment [7,8]. DNA and mRNA vaccines enable rapid design and production. DNA vaccines can induce cellular immunity, and mRNA vaccines can elicit both humoral and cellular responses depending on formulation and antigen design. However, DNA vaccines carry a theoretical risk of genomic integration, and mRNA vaccines face stability and delivery challenges; both may require multiple doses [9,10].

Given these considerations, recombinant viral vector vaccines have emerged as a promising alternative to the previously mentioned vaccine platforms. They use the natural infection machinery of a benign or attenuated virus to deliver and express foreign antigens within host cells. This platform offers several key advantages: it safely induces comprehensive humoral and cellular immune responses, often without exogenous adjuvants [1], and it allows for the presentation of antigens in their native conformation. The selection of an optimal viral vector is guided by several important criteria: (1) target host tropism, safety, permissive cell availability, route compatibility, and field epidemiology; (2) a genome with non-essential regions that can be replaced with foreign genes without impairing viral replication; (3) a high capacity for stable insertion and retention of foreign genetic material; and (4) the ability to drive high-level expression of the inserted antigen to elicit protective immunity.

Bovine herpesvirus type 1 (BoHV-1) is a cofactor of the bovine respiratory disease complex (BRDC), which in the US costs the cattle industry approximately $540 million in direct costs and an additional $5 billion in indirect losses each year [11,12]. Pseudorabies virus (PRV) inflicts substantial, multibillion-dollar annual economic losses on the global swine industry due to its high resistance, severe reproductive failures in sows, and high mortality rates in piglets [13]. The estimated economic impact of Marek’s disease herpesvirus (MDV) prevention measures on the global poultry industry exceeds US$1 billion annually due to carcass condemnation, reduced egg production, and vaccination program costs [14]. Equine herpesvirus type 1 (EHV-1) is widespread in horse populations. It is associated with substantial economic impact owing to outbreaks of respiratory disease, late-term abortions, and the often-fatal neurological syndrome equine herpes myeloencephalopathy [15]. Duck enteritis virus (DEV) causes an acute, highly contagious disease in ducks, geese, and swans. In immunologically naive migratory waterfowl, outbreaks at a single wintering site can result in mortality rates of up to 40% [16].

Over the past two decades, Alphaherpesviruses have proven to be particularly effective vectors for delivering multiple target epitopes (Table 1). Characterized by large, double-stranded DNA genomes (approximately 125–295 kbp) encoding around 70 open reading frames (ORFs), the genomes offer ample regions for genetic manipulation. The genomes contain non-essential or hypothetical genes that could be deleted or replaced to accommodate foreign antigen genes. Additionally, their broad tropism for mammals, birds, and reptiles makes these viruses versatile vaccine platforms. Prominent examples of Alphaherpesviruses currently used as vaccine vectors include: Bovine herpesvirus type 1 (BoHV-1), Pseudorabies virus (PRV), Marek’s disease virus (MDV), Equine herpesvirus type 1 (EHV-1), and Duck enteritis virus (DEV).

This review examines the current state of the art in using these herpesviruses as engineered vectors for vaccine development against major animal diseases, emphasizing their construction, efficacy, and potential for multivalent vaccines.

## 2. Literature Search Methodology

This review is a narrative synthesis of the published literature on alphaherpesvirus-vectored vaccines for veterinary applications; it is not a PRISMA-compliant systematic review or meta-analysis. To ensure transparency and reproducibility, a structured literature search was performed in PubMed, Google Scholar, and Web of Science (1990–2026) using keywords: “Bovine herpesvirus type 1” OR “BoHV-1” OR “BHV-1” OR “Pseudorabies virus” OR “PRV” OR “Suid herpesvirus 1” OR “Marek’s disease virus” OR “MDV” OR “Gallid alphaherpesvirus 2” OR “Equine herpesvirus type 1” OR “EHV-1” OR “Duck enteritis virus” OR “DEV” OR “*Mardivirus anatidalpha1*” OR “turkey herpesvirus” OR “HVT” AND “recombinant vaccine” OR “viral vector” OR “vectored vaccine” OR “DIVA” OR “challenge study”. Additional terms included “herpesvirus vector”, “foreign gene expression”, “multivalent vaccine”, and “pre-existing immunity”. The last search was performed on 1 June 2026. The full database-specific search strings, including Boolean operators and field tags, are provided in Supplementary Material S1.

The search retrieved 1247 records after deduplication. Titles and abstracts were screened for relevance to recombinant alphaherpesvirus vectors expressing heterologous antigens in target or model animal species. Inclusion criteria were: (1) original research articles describing the construction, immunogenicity, or efficacy of herpesvirus-vectored vaccines in susceptible animals or relevant models; (2) peer-reviewed reviews or meta-analyses (2010–2026) used for contextual background. Exclusion criteria were: non-English articles, non-peer-reviewed sources (conference abstracts, preprints), and studies lacking molecular or clinical relevance (e.g., purely diagnostic or epidemiological studies without vaccine evaluation).

After screening, 182 full-text articles were assessed, and 121 were included in this review (original research, *n* = 82; reviews/meta-analyses, *n* = 39). Reference chaining from identified articles yielded an additional 23 relevant papers. The narrative synthesis focuses on five alphaherpesvirus platforms (BoHV-1, PRV, MDV, EHV-1, DEV) for which sufficient peer-reviewed efficacy data exist. The limitation of our search methodology is the exclusion of non-English literature, because it may introduce language bias.

## 3. Herpesvirus Replication Cycle, Latency, Reactivation, Shedding, and Neuronal Transport

Herpesviruses are enveloped, double-stranded DNA viruses characterized by their biphasic cycle of lytic productive replication and lifelong latency [18]. This cycle is fundamental to the pathogenesis and persistence of the Herpesviridae family, which infects a wide range of vertebrates, including mammals, birds, reptiles, amphibians, and fish [19].

### 3.1. The Lytic Productive Replication Phase

Primary infection or reactivation from latency initiates the lytic replication cycle, culminating in the production of new viral progeny and, typically, the death of the infected cell. This occurs through the following steps:

1. Entry and gene expression: The cycle begins with viral entry into a permissive host cell, often mediated by specific interactions between viral glycoproteins and host cell receptors. Following entry and uncoating, the viral genome is transported to the nucleus. Viral gene expression occurs within a tightly regulated, sequential cascade. Initially, the immediate-early (alpha) genes are transcribed and translated into primarily regulatory proteins. This is followed by the early (beta) genes, involved in genome replication, and lastly, the late (gamma) genes, which encode structural components for virion assembly, are expressed [20,21].
2. Genome replication and assembly: Viral DNA replication occurs in the nucleus using a combination of viral and host enzymes. The newly synthesized genomes are packaged into pre-formed capsids, called the nucleocapsids. The latter acquires an initial envelope by budding through the inner nuclear membrane into the perinuclear space [22,23].
3. Egress and maturation: The newly acquired envelope is removed from the nucleocapsid particles in the perinuclear space by fusing with the outer nuclear membrane, releasing naked nucleocapsids into the cytoplasm. Final virion maturation involves a secondary envelopment process at cytoplasmic organelles, such as the trans-Golgi network or endosomes, where tegument proteins are added, and the nucleocapsid acquires its final envelope containing essential viral glycoproteins. Mature virions are released from the cell via exocytosis [23].

### 3.2. Establishment of the Latency Phase and Subsequent Reactivation

**Figure 1:**
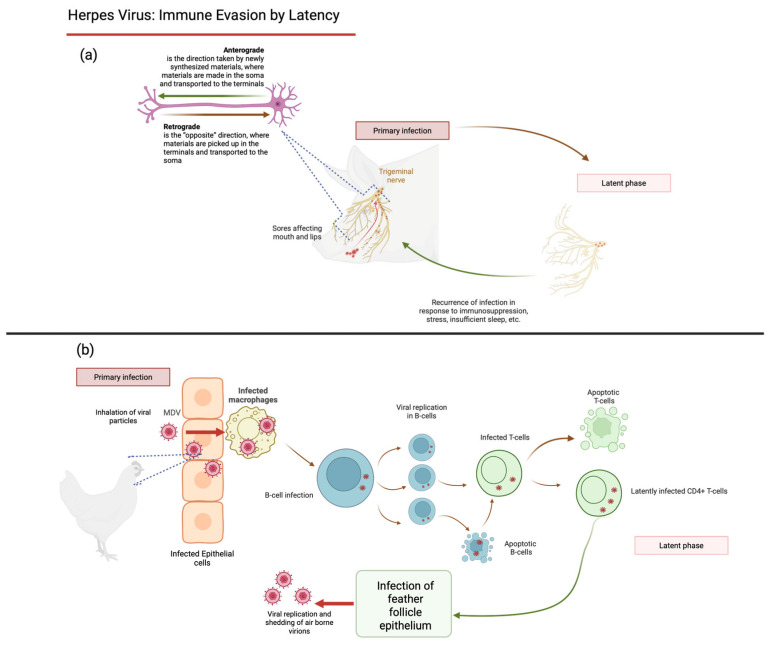
Mechanisms of Immune Evasion and Latency in Alphaherpesviruses. (**a**) Alphaherpesvirus in animals: Primary infection leads to lesions on the mouth and lips, followed by retrograde transport via the trigeminal nerve to neuronal cell bodies for the latent phase. Recurrence is triggered by immunosuppression, stress, or lack of sleep through anterograde transport back to epithelial tissues. (**b**) Marek’s Disease Virus (MDV) in Chickens: Inhalation of viral particles leads to infection of epithelial cells and macrophages. The virus subsequently infects B-cells and T-cells, establishing latency specifically in CD4+ T-cells while inducing apoptosis in non-latent lymphocytes. Continuous viral replication and shedding occur through the feather follicle epithelium. Figure generated by authors using BioRender Premium (Created in BioRender. Volchkov, P. (2026) https://BioRender.com/rma3hbs, accessed on 21 June 2026, agreement number SF29TH1E3H).

A defining feature of herpesviruses is their ability to establish a non-productive, latent infection following the initial lytic replication (Figure 1).

1. Latency: During latency, the viral genome persists as an episome in the nucleus of specific cell types with minimal viral gene expression. Viral gene expression is often limited to non-coding RNAs, such as microRNAs, as well as latency-associated transcripts (LATs). These products are crucial for maintaining the viral genome, suppressing lytic gene expression, and evading host immune surveillance. For example, Marek’s disease virus (MDV) establishes latency in chicken T lymphocytes, which can lead to transformation and tumorigenesis [24]. Similarly, human and animal alphaherpesviruses, such as Herpes Simplex Virus (HSV) and Equine Herpesvirus 1 (EHV-1), establish latency in the sensory neurons of their respective hosts [19].
2. Reactivation: Latent virus can periodically reactivate in response to various stressors (e.g., immunosuppression, fever, UV light), re-entering the lytic replication cycle. Reactivation involves the expression of immediate-early genes, leading to the full lytic cascade and production of infectious virus [25]. Research models are crucial for studying this switch. For instance, Tien et al. (2023) developed a fluorescent MDV-transformed cell line in which reactivation could be efficiently induced by lowering the temperature, providing a valuable ex vivo tool to study this process without the cytotoxicity associated with chemical inducers [26].

### 3.3. Retrograde and Anterograde Axonal Transport (In Alphaherpesviruses)

The ability to traffic within the intricate neuronal network is essential for neurotropic alphaherpesviruses such as EHV-1, Feline Herpesvirus 1 (FeHV-1), and Pseudorabies Virus (PRV) to establish latency in sensory ganglia and to spread to mucosal sites upon reactivation. This occurs via two directional pathways along the axon:

1. Retrograde transport, towards the cell body: During primary infection at a peripheral site (e.g., nasal mucosa or skin), viral capsids, often lacking a full envelope, are internalized at nerve termini. They are then transported along microtubules in a retrograde direction (from the axon tip to the neuronal cell body) using the host’s dynein motor complex. This journey delivers the viral genome to the neuron’s nucleus in the sensory ganglion (e.g., the trigeminal ganglion), where latency is established [27].
2. Anterograde transport towards the periphery: Upon reactivation from latency in the neuronal nucleus, newly assembled viral particles must travel back down the axon to the original site of infection or to other peripheral sites, thereby causing recurrent disease and facilitating transmission. Anterograde transport from the cell body to the axon terminal is more complex and can occur via two main models [28]:
  - Separate transport model: Naked virus capsids are transported down the axon, where they acquire a final envelope containing glycoproteins at the axon terminal. This is performed using vesicles derived from the trans-Golgi network that have been transported separately.
  - “Married” transport model: Fully enveloped virions are transported down the axon within membrane-bound vesicles.

The specific mechanisms and viral proteins governing these processes, such as the Us3 protein kinase mentioned in FeHV-1 studies by Ferrara et al. (2023), are active areas of research, as they represent prime targets for interventions aimed at preventing recurrent disease [29].

In conclusion, the herpesvirus life cycle encompasses lytic replication, the establishment of latency, and, in the case of neurotropic viruses, sophisticated bidirectional axonal transport systems. This demonstrates a high level of adaptation to the host. Understanding these interconnected processes, as highlighted by ongoing research on diverse animal herpesviruses [19], is fundamental to deciphering pathogenesis, developing models such as MDV-transformed cell lines [26], and ultimately designing strategies to control herpesviral diseases across species.

## 4. Host Immune Evasion Strategies of Herpesviruses

Herpesviruses have co-evolved with their hosts over millions of years, resulting in the development of complicated mechanisms to evade the host immune response and establish lifelong latent infections. A primary target of these evasion strategies is the Major Histocompatibility Complex (MHC) class I antigen presentation pathway, which is essential for cytotoxic T lymphocyte (CTL) recognition and clearance of virus-infected cells.

### 4.1. Global Suppression of Host Protein Synthesis Machinery

Many herpesviruses evade the immune system by broadly suppressing host gene expression. Alphaherpesviruses, such as Herpes Simplex Virus (HSV), deliver the virion host shutoff (vhs) protein (UL41), which degrades host mRNAs [30]. This protein is highly conserved across alphaherpesvirus species, including BoHV-1, PRV, EHV-1, and MDV [31]. The vhs protein functions as a nuclease, selectively degrading both host and viral mRNAs, thereby allowing the virus to suppress antigen presentation while permitting expression of viral immune-evasion proteins [32]. Similarly, certain gammaherpesviruses, such as Epstein–Barr virus (EBV), encode the alkaline exonuclease BGLF5, which also functions as an RNase to degrade host mRNAs, in conjunction with the previously mentioned MHC class I molecules [33,34]. This global host shutdown not only reduces antigen presentation but also impairs interferon responses, and CD4+ T cells help by limiting MHC class II synthesis [33,35].

### 4.2. Inhibition of Antigen Processing and Presentation

Herpesviruses employ multiple tactics to specifically disrupt the MHC class I pathway at nearly every stage. These strategies include:

1. Proteasome inhibition. Viruses can interfere with the synthesis of antigenic peptides through different mechanisms. Alphaherpesviruses like PRV encode the E3 ubiquitin ligase EP0 (also known as ICP0 in HSV-1), which promotes the proteasomal degradation of host antiviral proteins to subvert the host’s intrinsic and innate immune responses [36,37]. BoHV-1 and other alphaherpesviruses similarly encode proteins that either block immunoproteasome formation or prevent proper peptide generation, thereby limiting the pool of viral epitopes available for MHC class I presentation [38]. Betaherpesviruses such as murine cytomegalovirus (MCMV) encode the protein M27, which in turn blocks interferon-γ signaling, preventing the formation of the immunoproteasome [39]. Other viruses, such as EBV, produce proteins, such as EBNA-1, that contain glycine-alanine repeats (GArs) to inhibit their own proteasomal degradation, thereby limiting the pool of viral epitopes [40,41].
2. Peptide transport blockade: The Transporter Associated with Antigen Processing (TAP) is a common viral target. Several animal herpesviruses, including Bovine Herpesvirus 1 (BoHV-1), Equine Herpesviruses 1 and 4 (EHV-1, EHV-4), and Pseudorabies Virus (PRV), encode the UL49.5 protein, which inhibits TAP function by arresting it in a translocation-incompetent state or inducing its degradation [42]. BoHV-1 UL49.5 has been particularly well-characterized, showing potent inhibition of peptide transport across the ER membrane [43]. Marek’s Disease Virus similarly encodes homologous proteins (MDV012) that interfere with TAP-mediated peptide transport, preventing the loading of antigenic peptides onto MHC class I molecules [44]. Human Cytomegalovirus (HCMV) US6 also inhibits TAP by preventing ATP binding [45].
3. Retention and degradation of MHC I molecules: Herpesviruses can prevent MHC I molecules from reaching the cell surface through multiple mechanisms within the endoplasmic reticulum (ER). Equine Herpesvirus 1 encodes the pUL56 protein, which interferes with MHC class I assembly, resulting in retention of improperly folded molecules in the ER lumen [46]. While the pUL49.5 proteins encoded by PRV and BoHV-1 are generally classified as TAP transport inhibitors, their actions can cause a major secondary retention effect in the ER [47]. HCMV proteins US2 and US11 co-opt the host Endoplasmic Reticulum-Associated Degradation (ERAD) pathway to dislocate newly synthesized MHC I heavy chains from the ER to the cytosol for proteasomal destruction [48]. Similarly, Murine Gammaherpesvirus 68 (MHV-68) mK3, a viral E3 ubiquitin ligase, targets MHC I for ERAD [49]. Other mechanisms include the retention of mature complexes in the Golgi apparatus by Varicella-Zoster Virus (VZV) ORF66 [50].
4. Surface Downregulation: For MHC I molecules that do reach the plasma membrane, herpesviruses encode specialized factors to clear them from the cell surface. Among alphaherpesviruses, Equine Herpesvirus 1 (EHV-1) encodes the membrane protein pUL56, which enhances the endocytosis of cell surface MHC I molecules, directing them toward dynamin-dependent internalization and subsequent lysosomal degradation. In Pseudorabies Virus (PRV), surface clearance can be driven by early proteins or accelerated during active infection via antibody-induced cross-linking and passive co-internalization of MHC I complexes along with viral envelope glycoproteins like gB and gD [51]. These pathways differ from those seen with gammaherpesviruses like Kaposi’s Sarcoma-Associated Herpesvirus (KSHV), which encode the E3 ligases K3 and K5 (also known as MIR1 and MIR2), which ubiquitinate MHC I, inducing endocytosis and lysosomal degradation [52]. EBV protein BILF1 also promotes MHC I endocytosis and degradation via a ubiquitin-independent mechanism [53]. These surface downregulation mechanisms are critical for protecting infected cells from CTL recognition, as they eliminate the primary source of antigen presentation.

### 4.3. Evasion of Natural Killer (NK) Cellular Responses

Downregulation of MHC class I molecules increases the risk of infected cells becoming targets for NK cells. Herpesviruses counteract this by one of the following processes.

1. Selective downregulation: While certain human herpesviruses, such as HCMV, selectively decrease specific MHC I alleles, thereby downregulating HLA-A and HLA-B while leaving the NK inhibitory allele (HLA-E) intact to suppress NK cells, animal alphaherpesviruses typically deploy a non-selective, global shutdown of the classical MHC class I machinery [54].
2. Encoding MHC I homologs: HCMV encodes UL18, a functional homolog of MHC I that engages the inhibitory receptor LIR-1 on NK cells, providing a “false” inhibitory signal [55].
3. Targeting NK cell-activating ligands: Alphaherpesviruses downregulate stress-induced host ligands to prevent activation of NK cell receptors on infected surfaces. While human alphaherpesviruses target primate-specific NKG2D ligands like MICA and MICB, veterinary alphaherpesviruses target host-specific equivalents; for instance, Pseudorabies Virus (PRV) causes a rapid downregulation of the porcine NKG2D ligand pULBP1 and suppresses pMIC2 transcripts at the mRNA level [56]. Concurrently, PRV and BoHV-1 utilize their envelope glycoprotein D (gD) to downregulate CD112, a key ligand for the major activating NK cell receptor DNAM-1 [57]. EHV-1 similarly downregulates surface-expressed equine-specific activating ligands. By reducing the “eat me” signals on infected cells, these viruses effectively hide from NK cell recognition [58].
4. Viral microRNA (miRNA) Interference: Herpesviruses also use non-coding miRNAs to fine-tune immune evasion without activating a protein-mediated host interferon response. For example, Marek’s Disease Virus, a highly oncogenic alphaherpesvirus affecting chickens, generates viral miRNAs known as mdv1-miR-M4-5p, which act as a functional ortholog to host miR-155 to target host transcripts associated with T-cell activation and apoptotic pathways [59]. In PRV infection, host miR-21 is dramatically upregulated and directly suppresses the host chemokine CXCL10 (IP-10). Since CXCL10 is a powerful antiviral chemokine, the virus drives its post-transcriptional downregulation to cripple host innate signaling and local leukocyte recruitment [60]. Similarly, bovine herpesvirus 1 (BoHV-1) uses miRNA regulation of immune genes to reduce inflammation around infected cells. Furthermore, Beta- and gammaherpesvirus miRNAs, such as HCMV-miR-UL112, EBV miR-BART2-5p, and KSHV miR-K12-7, target MICB mRNA for degradation, reducing the “eat me” signal for NK cells [61,62].

Herpesviruses dedicate a significant portion of their genomes to multifunctional immune evasion that targets the host immune system at multiple levels. The convergent evolution of these strategies across alpha-, beta-, and gammaherpesviruses, including those infecting animal species like cattle (BoHV-1), horses (EHV-1/4), and poultry (Marek’s disease virus), underscores the critical importance of MHC I pathway disruption for viral persistence. This intricate molecular arms race not only ensures viral survival but also provides valuable insights into basic cell biology and potential therapeutic strategies for immune modulation [63]. The immune evasion mechanisms described above have important implications for recombinant vector design. For vaccine development, key viral genes responsible for immune evasion (e.g., gE, gI, UL49.5, ICP0 orthologues) are often deleted to enhance antigen presentation, improve safety, and enable DIVA strategies, while preserving the vector’s ability to drive protective immunity against the target pathogen. Additional details and descriptions are provided for the following herpesviruses.

## 5. Application of Herpesviruses in Animal Vaccinology

Compared with other viral vectors commonly used in veterinary vaccinology, such as adenovirus, poxvirus, or vesicular stomatitis virus, alphaherpesviruses offer distinct advantages: (i) larger genome capacity (up to ~295 kbp vs. ~36 kbp for adenovirus), allowing insertion of multiple or large foreign genes; (ii) the genome encodes several non-essential genes that can be replaced with genes of interest; (iii) natural neurotropism and ability to establish latency, which may prolong antigen expression; (iv) established DIVA compatibility through gene deletions; and (v) proven safety as live attenuated vaccines in target species for decades. However, challenges such as pre-existing immunity and potential reactivation remain.

### 5.1. Bovine Herpesvirus Type 1 (BoHV-1)

Bovine herpesvirus type 1 (BoHV-1; ICTV 2025 (International Committee on Taxonomy of Viruses (ICTV). Virus Taxonomy: 2025 Release. Available online: https://ictv.global/taxonomy (accessed on 21 June 2026)): *Varicellovirus bovinealpha1*) is a neurotropic pathogen responsible for infectious bovine rhinotracheitis, a disease associated with respiratory, ocular, and genital tract infections [64,65]. In adult cattle, clinical signs may be mild but often result in reduced milk yield, impaired fertility, and abortions. Importantly, BoHV-1 infection increases susceptibility to secondary bacterial pneumonias caused by pathogens such as *Mannheimia haemolytica* and *Histophilus somni*, leading to elevated morbidity and mortality. Following acute infection, the virus establishes lifelong latency within sensory neurons of the trigeminal ganglia. It can be reactivated and shed by asymptomatic carriers under stress, facilitating viral spread through direct contact, frozen semen, and embryos.

BoHV-1 as a conventional pathogen vaccine. Conventional vaccines against BoHV-1 include modified live virus (MLV) and inactivated formulations. MLV vaccines induce strong humoral and cellular immunity but can establish latency and potentially reactivate. In the European Union, control strategies have shifted toward the use of gene-deleted vaccines, particularly those lacking the glycoprotein E (gE) gene, which enable differentiation between infected and vaccinated animals (DIVA strategy) [11,66]. For example, ⁠BoHV-1 gE-deleted vaccines (such as Bovilis^®^ IBR Marker Live, Intervet International B.V. (MSD Animal Health), Boxmeer, The Netherlands) allow serological distinction between vaccinated animals and those naturally infected with wild-type BoHV-1, which expresses gE. This DIVA strategy is critical for eradication programs. In other countries, multivalent formulations combining BoHV-1 with bovine viral diarrhea virus (BVDV) and bovine respiratory syncytial virus (BRSV) are commonly administered [67].

BoHV-1 as a vector for heterologous antigens. Beyond its role as a vaccine against BoHV-1, BoHV-1 has been developed as a versatile platform for delivering foreign antigens from other pathogens. Its large genome (∼135 kbp), well-characterized non-essential gene loci (e.g., gE, gG, gIII (known now as glycoprotein C-gC), UL41), and ability to stably express heterologous proteins without compromising viral replication make it an attractive vector. Early work by Kit et al. (1991) demonstrated the feasibility of this approach, in which a recombinant BoHV-1 was engineered to express a foot-and-mouth disease virus (FMDV) VP1 epitope fused to the viral glycoprotein gC [68]. This construct successfully elicited protective anti-FMDV antibodies in calves and conferred immunity against a virulent BoHV-1 challenge, primarily through a humoral immune response [68].

Subsequent studies have expanded the utility of BoHV-1 vectors for other pathogens; for example, recombinants expressing the E2 glycoprotein of bovine viral diarrhea virus (BVDV) were developed, resulting in efficient incorporation of the heterologous protein into the BoHV-1 envelope. Although this incorporation modestly affected viral entry and egress in vitro, the platform proved promising for the development of both live- and inactivated-BoHV-1-vectored BVDV vaccines [69].

Further innovation was demonstrated by Ren et al. (2009) who inserted a synthetic FMDV VP1 gene into the gE locus of BoHV-1 under the control of a human cytomegalovirus promoter [70]. The resulting recombinant, BoHV-1/gE−/VP1, induced significant virus-neutralizing antibodies in a rabbit model, highlighting its potential as a bivalent vaccine against FMD and infectious bovine rhinotracheitis (IBR) [70].

Recently, a quadruple-gene-mutated BoHV-1 vector (BoHV-1 QMV) was engineered to co-express the BVDV type 2 E2 and Erns proteins fused to bovine GM-CSF [71]. In a comparative calf study, this prototype subunit vaccine elicited robust neutralizing antibody responses against both BoHV-1 and BVDV-2 and induced cross-reactive cellular immunity. Following a virulent BVDV-2 challenge, vaccinated calves showed a rapid, robust recall of neutralizing antibodies and superior cellular immune responses compared with calves vaccinated with a commercial multivalent vaccine [71].

BoHV-1 has also been utilized as a vaccine against other diseases. Using CRISPR-Cas9 and homologous recombination, a BoHV-1 vector was constructed to express the rabies virus glycoprotein (RABV G) in place of its gE gene. The recombinant virus, BoHV-1-ΔgE-G, stably expressed RABV G on the virion surface and induced protective levels of virus-neutralizing antibodies in both mice and cattle after a single immunization, without causing clinical symptoms. This study underscores the potential of BoHV-1 as a vector for preventing zoonotic diseases in cattle [72].

Collectively, these advances illustrate the adaptability and efficacy of BoHV-1 as a recombinant vaccine vector capable of delivering protective antigens against multiple economically significant animal pathogens.

### 5.2. Pseudorabies Virus (PRV)

Pseudorabies virus (PRV; ICTV 2025: *Varicellovirus suidalpha1*) is a neurotropic alphaherpesvirus and the etiological agent of Aujeszky’s disease in swine [73]. The disease is characterized by a range of clinical manifestations, including respiratory distress, neurological disorders, and reproductive failure, leading to substantial economic losses in the global swine industry. Significant efforts have therefore been devoted to developing effective vaccines and control programs.

Classical Bartha-K61 vaccine and its use as a vector backbone. Among the most widely studied and utilized vaccine strains is the attenuated Bartha-K61 strain, developed by Adorján Bartha in 1961 through serial passaging in various cell lines [74,75]. This strain has become a cornerstone not only as an effective monovalent vaccine but also as a well-characterized molecular backbone for developing recombinant vector vaccines. Its genomic stability, safety profile, and capacity for foreign gene insertion make it an ideal platform for antigen delivery.

The Bartha-K61 backbone has been successfully engineered to express heterologous antigens from other swine pathogens. For example, a recombinant PRV expressing the GP5 protein of porcine reproductive and respiratory syndrome virus (PRRSV) conferred significant clinical protection and reduced pathological lesions in piglets following PRRSV challenge [76]. Similarly, a Bartha-K61 vector expressing the hemagglutinin (HA) of swine influenza virus (H3N2) protected mice against lethal challenge [77], and recombinants expressing HA or neuraminidase of pandemic H1N1 influenza virus inhibited viral replication in pigs [78]. These examples demonstrate the versatility of classical Bartha-K61 as a flexible vector platform for multivalent vaccines.

Emergence of PRV variants and variant-adapted vectors. Beginning around 2011, novel PRV variants with distinct antigenic profiles emerged in China, causing severe outbreaks even in pigs vaccinated with classical Bartha-K61 [79]. These variants carry mutations in immunodominant glycoproteins (gB, gC, gD) that allow escape from Bartha-K61-induced immunity. In response, researchers have developed optimized vaccine candidates by replacing key immunogenic genes (e.g., gB, gD, gC) in the Bartha backbone with their counterparts from circulating Chinese variant strains [79]. For example, a gD and gC-substituted PRV vaccine strain provided complete clinical protection and reduced viral shedding against a Chinese PRV variant [80]. Similarly, swapping gB from a variant into the Bartha backbone improved immunogenicity against heterologous challenge [81]. These variant-adapted constructs are at various stages of preclinical and clinical evaluation, though none have yet fully replaced Bartha-K61 in commercial markets outside China.

DIVA compatibility and diagnostic aspects. The Bartha-K61 strain naturally lacks gE and gI due to deletions acquired during attenuation. This enables DIVA serological differentiation: vaccinated animals remain antibody-negative for gE, whereas field-infected animals are gE-positive. Most variant-adapted vectors retain these deletions, preserving DIVA compatibility. However, diagnostic tests must be periodically validated against circulating variant strains to ensure continued sensitivity.

Safety and zoonotic considerations. While PRV has long been considered a veterinary pathogen restricted to pigs, novel variants since 2017 have been shown to breach species barriers, causing 31 laboratory-confirmed human cases of encephalitis and endophthalmitis in China by 2023, with high mortality and disability rates [82]. This zoonotic spillover risk underscores the need for rigorous host-range evaluation, enhanced biosecurity, and careful risk–benefit assessment for any live PRV-vectored vaccine, particularly in regions with high pig density and potential human exposure. Deletion of additional virulence genes (e.g., TK) and use of non-replicating or disabled infectious single-cycle (DISC) designs may further improve safety.

In summary, the classical PRV Bartha-K61 backbone remains a robust and flexible vector platform. However, the emergence of antigenically distinct variants has driven the development of variant-adapted vectors tailored to regional epizootiology. Future efforts should focus on balancing broad cross-protection, DIVA compatibility, and safety, with particular attention to zoonotic risks.

### 5.3. Marek’s Disease Virus (MDV)

Marek’s Disease Virus (MDV) is the causative agent of Marek’s Disease (MD), a highly contagious, lymphoproliferative disorder in chickens characterized by T-cell lymphoma formation, peripheral nerve enlargement, visceral organ infiltration, and severe immunosuppression. MDVs belong to the genus *Mardivirus* within the *Alphaherpesvirinae* subfamily and are classified into three serotypes: *Mardivirus gallidalpha2* (formerly GaHV-2 or MDV-1), which includes pathogenic and oncogenic strains; *Mardivirus gallidalpha3* (formerly GaHV-3 or MDV-2), comprising non-oncogenic chicken viruses; and *Mardivirus meleagridalpha1* (formerly MeHV-1 or MDV-3/HVT), the non-oncogenic turkey herpesvirus [83].

MDV-1-based vaccines have long been used successfully in poultry due to their ability to confer lifelong protection, a safe profile, compatibility with in ovo or day-old administration, and the capacity to overcome maternal antibodies. These attributes, combined with the virus’s ability to persist in the host and co-administer with other serotypes, make MDV-1 an excellent candidate not only for protecting against MD but also for serving as a recombinant vector against other avian pathogens [84,85].

A compelling example is the development of an MDV-1 vector (rMDV-VP2) expressing the VP2 capsid protein of Infectious Bursal Disease Virus (IBDV) [86]. In dose-dependent trials, a single administration of 1000 PFU fully protected chickens against virulent IBDV challenge, while lower doses provided 80–90% protection. Notably, in commercial layers with maternal antibodies, rMDV-VP2 achieved 90% protection, which is superior to that of a conventional live IBDV vaccine, while also conferring 95–100% protection against a very virulent MDV-1 challenge [86].

MDV vectors have also been engineered against avian influenza. A recombinant MDV-1 (rMDV-HA) expressing the hemagglutinin (HA) gene of highly pathogenic avian influenza virus (HPAIV) H5N1 induced 80% protection against HPAIV challenge and complete protection against lethal MDV in immunized chickens [87].

Another significant target is Avian Leukosis Virus subgroup J (ALV-J), an immunosuppressive retrovirus that causes substantial economic losses and for which no commercial vaccine currently exists. Two recombinant MDV-1 constructs were generated: one expressing the ALV-J env gene (rMDV/ALV-env) and another expressing both gag and env (rMDV/ALV − gag + env). Both recombinants expressed the intended antigens without impairing viral growth in vitro. In vaccination-challenge studies, both constructs induced specific antibodies and reduced viremia rates in chickens challenged with ALV-J, with rMDV/ALV-env conferring slightly stronger protection [88].

MDV vectors have also been applied to control Infectious Laryngotracheitis (ILT), a severe respiratory disease of chickens. Traditional control relies on live attenuated ILT vaccines, but recombinant vectors, such as turkey herpesvirus (HVT), have also been used. However, HVT-based vaccines may offer limited protection against very virulent plus MDV (vv + MDV) strains. In contrast, MDV-1-based vectors, particularly those derived from the oncogenic Md5 strain with the MEQoncogene deleted (BACΔMEQ), have shown superior efficacy against vv + MDV. Two bivalent vaccines were developed using this backbone, BACΔMEQ-gB and BACΔMEQ-gJ, which express ILT virus glycoproteins B and J, respectively. Both were experimentally proven to be safe and showed high growth rates in commercial chickens with maternal antibodies. BACΔMEQ-gB protected as effectively as a commercial rHVT-LT vaccine against ILT challenge, while BACΔMEQ-gJ alone was less protective. Importantly, both MDV-1 recombinants provided better protection against an early challenge with the vv + MDV strain 648A than the commercial rHVT-LT or CVI988 vaccines [89].

These studies collectively emphasize the versatility and efficacy of MDV, particularly MDV-1, as a stable and immunogenic vector platform. Its ability to express foreign antigens from multiple avian pathogens, while retaining its own protective capacity against MD, makes it a powerful tool for developing multivalent vaccines that can simplify poultry vaccination programs and enhance flock health management.

### 5.4. Equine Herpesvirus Type 1 (EHV-1)

Equine herpesvirus type 1 (EHV-1; ICTV 2025: *Varicellovirus equidalpha1*) is an alphaherpesvirus of the genus *Varicellovirus* and a significant respiratory pathogen in equine populations worldwide [90,91]. Beyond respiratory illness, EHV-1 infection can lead to abortion in pregnant mares and neurological disease, contributing to substantial economic losses in the equine industry. The modified-live virus (MLV) vaccine strain RacH, attenuated through extensive passaging in swine kidney cells, is widely used in Europe and the United States for protection against EHV-1. This attenuation is attributed to a spontaneous deletion in gene 67 (IR6) and truncations in glycoprotein B, rendering it safe for use in horses and other species while retaining strong immunogenicity [92,93].

The RacH strain possesses several characteristics that make it an attractive viral vector platform. It efficiently infects a broad range of cell types from multiple species (including canine, feline, avian, and human cell lines), stably expresses foreign genes, induces both humoral and cellular immunity, and does not require adjuvants for efficacy [94,95,96]. Furthermore, its large genome provides ample capacity to insert heterologous genes without compromising viral replication, making it well-suited for the development of recombinant vector vaccines.

Early studies confirmed the utility of EHV-1 as a vector by demonstrating its ability to infect non-equine cell lines and to drive high-level expression of reporter genes, such as enhanced green fluorescent protein (EGFP), under the control of strong promoters, including the human cytomegalovirus immediate-early promoter [97]. Subsequent research expanded its application to the expression of protective antigens from other important pathogens.

A notable example is the construction of a recombinant EHV-1 expressing the prM and E structural proteins of West Nile virus (WNV) [96]. Horses vaccinated with this vector showed significant levels of WNV-neutralizing antibodies and WNV-specific IgG subtypes induced following a single dose, with durable responses observed throughout the study period. Control horses remained sero-negative, confirming the immunogenicity and specificity of the vectored vaccine [70].

EHV-1 has also been engineered to address influenza viruses in both equine and canine hosts. Following the emergence of canine influenza virus (CIV), which is closely related to the equine influenza virus (EIV) H3N8 subtype, researchers developed an EHV-1 vector expressing the H3 hemagglutinin of a contemporary EIV isolate [98]. This recombinant vaccine elicited robust influenza-specific immune responses in both mice and dogs following intranasal or subcutaneous administration. Importantly, vaccinated dogs challenged with a virulent CIV strain exhibited significantly reduced clinical signs and viral shedding compared with unvaccinated controls, demonstrating cross-protective efficacy and potential to reduce virus transmission.

Despite these promising attributes, significant safety concerns persist regarding the widely used RacH strain and the newly developed gene-deletion mutants (vToH-DMV and vToH-QMV) [99], as their attenuation and vector utility must be balanced against the inherent risks of alphaherpesvirus biology. While RacH is considered relatively safe due to its IR6 deletion and gB truncations, the more extensively modified quadruple-deletion mutant (vToH-QMV) lacks rigorous evaluation in its natural equine host for its ability to establish lifelong latency in neuronal tissues or to reactivate under immunosuppressive conditions [99]. Furthermore, quantitative PCR analyses have focused primarily on post-challenge clearance of wild-type virus rather than systematically characterizing the shedding profile of the vaccine viruses themselves following immunization, leaving the risk of horizontal transmission to unvaccinated or immunocompromised horses inadequately defined. There is also the unresolved ecological risk that these modified live viruses could recombine with circulating field strains, potentially restoring deleted genes (gE, UL43, and UL56) and leading to reversion to virulence, a scenario that current data leave entirely unaddressed. Until these critical safety gaps (latency, reactivation, shedding dynamics, and genomic stability) are systematically investigated in horses rather than mice, the translational application of RacH and its mutant derivatives as safe, deployable vector platforms remains highly speculative.

### 5.5. Duck Enteritis Virus (DEV)

Duck enteritis virus (DEV), also known as *Mardivirus anatidalpha1*, is the causative agent of Duck Viral Enteritis (DVE) or “duck plague.” This highly contagious and often fatal disease affects domestic and wild waterfowl, including ducks, geese, and swans, causing substantial economic losses in the poultry industry and threatening wild bird populations [100]. With a global distribution facilitated by migratory waterfowl, controlling DVE remains a significant challenge.

Current vaccination strategies primarily rely on live-attenuated and inactivated DEV vaccines, which are generally administered to birds at two weeks of age in various countries within endemic regions, particularly across Asia and Africa, the European Union, and the United States [101,102]. However, vaccine efficacy can be limited in birds with pre-existing immunity or during active outbreaks, and the chronic, shedding nature of DEV infection complicates disease management [103]. These limitations have spurred interest in utilizing attenuated DEV strains as recombinant vectors for multivalent vaccination.

The attenuated Chinese vaccine strain C-KCE has emerged as a particularly promising vector backbone. In pioneering work, researchers successfully incorporated the hemagglutinin (HA) gene from highly pathogenic avian influenza virus (HPAIV) H5N1 into the C-KCE genome using bacterial artificial chromosome (BAC) technology, creating the recombinant pBAC-C-KCE-HA [104,105,106]. This bivalent vaccine provided rapid and durable immunological protection in ducks and chickens against both DEV and H5N1 influenza, demonstrating the feasibility of DEV as a vector for avian influenza [107].

Similarly, DEV has been engineered to combat duck Tembusu virus (DTMUV), an emerging flavivirus affecting ducks and mammals. A recombinant C-KCE vector expressing the DTMUV envelope (E) protein (C-KCE-E) was constructed using a modified MAGIC strategy [108,109]. Immunised ducks developed high titers of DTMUV-neutralising antibodies, confirming the immunogenicity of the vectored antigen and underscoring the potential of DEV-based vaccines for dual protection in waterfowl [89].

The broad species tropism and genetic plasticity of DEV have further enabled its use in protecting chickens against non-waterfowl pathogens. Recombinant DEV vaccines expressing structural proteins of infectious bronchitis virus (IBV), including the nucleocapsid (N), spike (S), and S1 proteins, were developed and evaluated in chickens [110]. These constructs (rDEV-N, rDEV-S, and rDEV-S1) induced robust humoral and cellular immune responses, lowered the CD4+/CD8+ T-cell ratio, reduced viral shedding, and decreased mortality following challenge with a virulent IBV strain.

Recently, a recombinant DEV with a deletion in the UL2 gene was engineered to express the HA protein from a duck-origin H9N2 avian influenza virus (rDEV-ΔUL2-HA) [111]. A single low-dose immunisation in ducks elicited strong protective immunity against both lethal DEV challenge and H9N2 infection, significantly reducing H9N2 viral shedding and enhancing survival.

Although DEV-based vaccine vectors show promise, major biosafety concerns remain. Modified strains have not been fully evaluated in natural waterfowl populations for latency, reactivation, shedding, or long-term genetic stability, and the risk of transmission or recombination with field strains remains unclear. Until these issues are systematically studied in target bird hosts, their safe widespread field use remains uncertain.

## 6. Conclusions

The utilisation of animal herpesviruses as vectors for subunit vaccine development represents a significant and evolving frontier in veterinary vaccinology. As reviewed, alphaherpesviruses such as BoHV-1, PRV, MDV, EHV-1, and DEV possess intrinsic biological properties, including large, stable genomes, non-essential gene regions amenable to genetic manipulation, broad host tropisms, and the ability to induce robust and durable immune responses, that render them highly suitable as recombinant vaccine platforms.

Each vector system has demonstrated remarkable versatility and efficacy in expressing heterologous antigens from a diverse array of pathogens. From BoHV-1-based bivalent vaccines against FMD and BVDV to PRV-vectored constructs targeting swine influenza and PRRSV to MDV platforms conferring dual protection against IBDV and avian influenza, the proof of concept has been firmly established. Similarly, EHV-1 and DEV have shown promise in combating zoonotic threats such as West Nile virus and avian influenza, as well as addressing important species-specific diseases (Table 2).

The collective evidence emphasized several key advantages of herpesvirus-vectored vaccines:

1. Safety and Differentiation. Many vectors are derived from established, attenuated vaccine strains, offering a high safety profile. Gene-deletion strategies further enhance DIVA (Differentiating Infected from Vaccinated Animals) capabilities, which are crucial for disease surveillance and eradication programs.
2. Dual or Multivalent Protection. These platforms can deliver immunity against both the vector’s causative disease and one or more heterologous pathogens, simplifying vaccination regimens and reducing costs.
3. Comprehensive Immunity. They effectively stimulate both humoral and cellular immunity.

Several herpesvirus-vectored vaccines have already reached the market or advanced clinical trials in veterinary medicine. For example, recombinant HVT (turkey herpesvirus) vectors expressing antigens from IBDV, ILTV, and NDV are commercially available (e.g., VAXXITEK^®^ HVT + IBD (Boehringer Ingelheim Animal Health USA Inc., Gainesville, GA, USA), Poulvac^®^ Procerta HVT-IBD (Zoetis Inc., Parsippany, NJ, USA)). Additionally, in China, due to several PRV outbreaks, rather than licensing a simple Bartha-K61 + PRRSV GP5 construct, current Chinese patent applications and pipeline research are focused on multi-gene constructs (co-expressing PRRSV GP5 + Matrix (M) proteins to form virus-like particles) embedded into local, variant-based PRV backbones rather than the classical Bartha strain [112]. In Europe, BOHV-1 gE-deleted marker vaccines (e.g., Bovilis^®^ IBR Marker Live) are widely used. Despite these successes, many promising constructs described in this review remain at the experimental stage, with barriers including regulatory approval costs, manufacturing scalability, and pre-existing immunity in target populations.

Despite these advances, challenges remain, including optimizing antigen expression, ensuring genetic stability during manufacturing, addressing pre-existing vector immunity, and navigating regulatory requirements. Long-term field efficacy, cross-species safety, and environmental persistence also require study. Live herpesvirus vectors pose biosafety risks. For example, PRV variants have caused human encephalitis in China [82], and vaccine shedding could affect naive or pregnant animals. Latency is a double-edged sword; on the one hand, it may prolong antigen expression, but on the other hand, it can lead to reactivation and reversion to virulence. Moreover, deleting latency genes reduces risk but may lower immunogenicity. Recombination with wild-type or vaccine strains (notably PRV and MDV) could restore virulence; marker deletions mitigate this risk but do not eliminate it. Finally, large-scale production demands permissive cell lines and rigorous quality control. At the same time, regulatory agencies require proof of no recombination, no genomic integration, and long-term safety, which in turn adds substantial time and cost.

Looking ahead, the integration of CRISPR/Cas9-mediated precision editing, artificial intelligence-driven antigen design, and synthetic biology is expected to improve further the safety, efficacy, and versatility of herpesvirus vectors. Emphasis on mucosal delivery systems, thermostable formulations, and One Health-aligned vaccine strategies will be essential for effective field deployment in diverse animal populations. The development of broader multivalent or universal vaccine platforms, combined with molecular adjuvants to modulate immune responses, offers significant promise for next-generation veterinary vaccines.

In summary, herpesvirus-vectored subunit vaccines provide a robust, adaptable, and practical approach for controlling complex animal diseases. Leveraging the natural biology of these viruses, this platform has significant potential to improve animal health and welfare, strengthen food security, protect wildlife, and reduce the economic and public health impacts of zoonotic diseases. Ongoing research, interdisciplinary collaboration, and industry partnerships will be critical to advancing these vaccine candidates into widely adopted solutions for sustainable prevention and control of animal diseases.

## 7. Future Directions: Beyond Vaccines, the Potential as Gene Therapy Vectors

While this review focuses on the use of alphaherpesviruses as effective platforms for viral-vectored vaccines, an important consideration is their potential to deliver genes of interest to specific organs for gene therapy.

As mentioned previously, the same intrinsic features that make alphaherpesviruses effective vaccine platforms, i.e., large packaging capacity, stable episomal persistence, broad host tropism, and in many cases natural neurotropism, also position them as promising delivery vehicles for therapeutic gene therapy applications. These applications could benefit both veterinary medicine and, with appropriate adaptation, human medicine.

Extending this concept to human gene therapy, related alphaherpesviruses, most notably herpes simplex virus type 1 (HSV-1), have already entered clinical trials for usage in conditions such as malignant glioma, pain management, and inherited retinal diseases [113,114]. These human-directed HSV-1 applications serve as conceptual examples of herpesvirus engineering but are not directly transferable to the veterinary alphaherpesvirus vectors reviewed here; each veterinary platform requires independent evaluation of safety, efficacy, and regulatory approval for its intended species and field conditions. HSV-1 structure and function have been studied extensively, facilitating the design and engineering of HSV-1-derived vectors as gene-transfer vehicles for various treatment strategies in experimental gene-therapy models [115]. Combined selective oncolysis and modulation of the immune response mediated by replication-conditional, multiple-mutated HSV-1 vectors appears to be a highly promising approach in the battle against malignant glioma [113,115]. Helper-virus-free HSV/AAV hybrid amplicon vectors have great promise for mediating long-term gene expression in the PNS and CNS to treat neurodegenerative disorders, injuries, or chronic pain. The design of HSV-1-derived vectors, which are targeted to specific cell populations and support transcriptionally regulatable transgene expression, is a focus of current and future research [113,114,115,116]. The importance of developing multiple modes of mechanical vector delivery that ensure sufficient vector distribution cannot be overstated. Gene therapy is a multivariate approach, with each variable equally important to overall success in a clinical setting. Therefore, the design of efficient gene therapy protocols relies on the concerted research and interaction of basic and clinical scientists.

The engineering principles refined in veterinary viral-vector vaccines, such as deleting immune-evasion genes to enhance safety and persistence, are directly applicable to human-directed platforms. For instance, the deletion of the neurovirulence gene γ34.5 in HSV-1 yields replication-competent oncolytic viruses (e.g., talimogene laherparepvec, TVEC) approved for melanoma, while HSV-1 amplicon vectors can deliver large or multiple transgenes to neurons with minimal toxicity [117]. Importantly, the veterinary vectors reviewed here (PRV, BoHV-1, and EHV-1) also offer distinct advantages for human applications. PRV is inherently non-pathogenic in humans and has been used as a safe, high-capacity vector for neuronal circuit mapping, with potential to deliver gene therapies to specific neural populations [118,119,120]. BoHV-1 and EHV-1 can efficiently transduce human cell lines and, when rendered replication-defective, may serve as cost-effective, scalable alternatives to HSV-1 for ex vivo gene delivery or vaccine-based cancer immunotherapy [121,122]. However, these vectors should be used with extensive caution because reports of PRV, which was historically considered a veterinary pathogen restricted to pigs, have now emerged as a zoonotic agent capable of causing severe and often fatal infections in humans. Since 2017, novel pseudorabies virus variants in China have breached species barriers, leading to 31 laboratory-confirmed human cases of encephalitis and endophthalmitis with high mortality and disability rates by 2023 [82].

Thus, the engineering strategies, vector backbones, and safety concepts refined in veterinary vaccinology are directly applicable to the broader field of human gene therapy. The convergence of these disciplines underscores the versatility of herpesvirus platforms, not only for preventing infectious diseases but also for addressing some of the most challenging genetic and neoplastic conditions across species. Concrete One Health actions should include shared surveillance of vaccine-related adverse events across human and animal populations, harmonized regulatory frameworks for dual-use vectors, and biosecurity measures to prevent environmental shedding or spillover of recombinant viruses. While regulatory hurdles and manufacturing complexities remain, the cross-pollination between veterinary and human applications accelerates innovation and highlights the One Health relevance of these powerful viral vectors.

## Figures and Tables

**Table 1:** Advantages and challenges of Herpesvirus vectors compared to other vaccine platforms.

Vaccine Platform	Key Advantages	Major Limitations	Ideal Use Situation	References
Live Attenuated	Strong, long-lasting cellular & humoral immunity; mimics natural infection.	Risk of reversion, residual virulence; not suitable for immunocompromised hosts.	Endemic diseases in healthy populations where strong immunity is critical.	[5,6]
Inactivated	Safe; no risk of replication; stable.	Weaker cellular immunity; may require adjuvants; risk of epitope alteration during inactivation.	Where safety is paramount and humoral immunity is sufficient.	[3,4]
Subunit/virus like particles (VLP)	Safe; well-defined composition; no infectious material.	Poor immunogenicity; requires strong adjuvants & boosters; complex manufacturing.	Diseases where a single protein confers protection and safety is a priority.	[7,8]
Nucleic Acid (DNA/RNA)	Rapid design/production; strong cellular (DNA) or humoral (RNA) responses.	DNA: risk of genomic integration; RNA: stability/delivery challenges; potential inflammatory responses.	Emerging outbreaks; pathogens requiring rapid vaccine updates.	[9,10]
Herpesvirus Vectored	Strong humoral & cellular responses; single-dose potential; multivalent possible; stable genome; DIVA-capable.	Pre-existing immunity to vector may reduce efficacy; more complex regulatory pathway; limited by vector tropism.	Complex diseases requiring durable, broad immunity; eradication programs needing DIVA.	[17]

**Table 2:** Veterinary Herpesvirus vectored vaccine candidates.

Vector Platform	Inserted Antigen(s)	Target Pathogen/Disease	Animal Model	Immune Response Measured	Translational Status	Protection Efficacy (%)/Key Outcome	Reference (From Text)
BoHV-1	FMDV VP1 epitope fused to gIII	Foot-and-Mouth Disease Virus (FMDV)	Calves	Anti-FMDV antibodies	Proof-of-concept	Protective antibodies; immunity against virulent BoHV-1 challenge	[68]
BoHV-1	BVDV E2 glycoprotein	Bovine Viral Diarrhea Virus (BVDV)	(Not specified–in vitro)	E2 incorporation into envelope	Proof-of-concept	Promising platform for live/inactivated BVDV vaccines	[69]
BoHV-1	FMDV VP1 (O/China/99)	FMDV & Infectious Bovine Rhinotracheitis (IBR)	Rabbits	Virus-neutralizing antibodies	Proof-of-concept	Induced neutralizing antibodies; potential bivalent vaccine	[70]
BoHV-1 (QMV)	BVDV-2 E2 + Erns + GM-CSF	BVDV-2	Calves	Neutralizing Ab, cellular immunity	Advanced Preclinical	Superior Ab recall & cellular response vs. commercial vaccine	[71]
BoHV-1	Rabies virus glycoprotein (RABV G)	Rabies	Mice, Cattle	Virus-neutralizing antibodies (VNAs)	Advanced Preclinical	Single dose induced protective VNA; no clinical symptoms	[72]
PRV (Bartha)	PRRSV GP5	Porcine Reproductive & Respiratory Syndrome Virus (PRRSV)	Piglets	Clinical signs, pathological lesions	Advanced Preclinical	Reduced clinical signs & lesions post-challenge	[76]
PRV (Bartha)	Swine Influenza HA (H3N2)	Swine Influenza (H3N2)	Mice	HA-specific antibodies	Proof-of-concept	100% survival in mice post-lethal H3N2 challenge	[77]
PRV (Bartha)	Swine Influenza HA/NA (H1N1)	Pandemic H1N1 Influenza	Pigs	Viral replication inhibition	Advanced Preclinical	Inhibited viral replication in pigs	[78]
MDV-1	IBDV VP2	Infectious Bursal Disease Virus (IBDV)	Chickens (layers, with maternal Abs)	Seroconversion	Commercial Standard (Analogous to VAXXITEK^®^, Boehringer Ingelheim Animal Health USA Inc., Gainesville, GA, USA)	100% protection with 1000 PFU; superior to live IBDV vaccine	[86]
MDV-1	HPAIV HA (H5N1)	Highly Pathogenic Avian Influenza (H5N1)	Chickens	HA-specific antibodies	Advanced Preclinical	80% vs. HPAIV; 100% vs. MDV	[87]
MDV-1	ALV-J Env (±Gag)	Avian Leukosis Virus subgroup J (ALV-J)	Chickens	Antibodies, viremia rates	Advanced Preclinical	Reduced viremia; Env-only slightly more protective	[88]
MDV-1 (BACΔMEQ)	ILTV gB or gJ	Infectious Laryngotracheitis Virus (ILT) & MDV	Chickens (with maternal Abs)	Protection vs. ILT & vv + MDV	Advanced Preclinical	Good ILT protection; superior vv + MDV protection vs. rHVT-LT	[89]
EHV-1 (RacH)	WNV prM/E	West Nile Virus (WNV)	Horses	WNV-neutralizing antibodies, IgG subtypes	Advanced Preclinical	Induced neutralizing Abs after single dose	[96]
EHV-1 (RacH)	EIV H3 hemagglutinin	Canine/Equine Influenza (H3N8)	Dogs, Mice	Clinical signs, viral shedding	Advanced Preclinical	Reduced signs & shedding in dogs post-CIV challenge	[98]
DEV (C-KCE)	HPAIV HA (H5N1)	H5N1 Avian Influenza & Duck Viral Enteritis (DVE)	Ducks, Chickens	Protection, immunological response	Advanced Preclinical	Rapid & long-term protection against both pathogens	[105,107]
DEV (C-KCE)	DTMUV E protein	Duck Tembusu Virus (DTMUV)	Ducks	DTMUV-neutralizing antibodies	Advanced Preclinical	Induced neutralizing Abs; potential bivalent vaccine	[108,109]
DEV	IBV N, S, S1 proteins	Infectious Bronchitis Virus (IBV)	Chickens	Humoral & cellular immunity, viral shedding, mortality	Proof-of-concept	Reduced shedding & mortality; improved cellular immunity	[86]
DEV	LPAIV HA (H9N2)	H9N2 Avian Influenza & DVE	Ducks	Protection, viral shedding	Advanced Preclinical	Single low dose protected against lethal DEV & H9N2; reduced shedding	[111]

## Data Availability

This is a review article; no new data or code were generated or analyzed in this study.

## Supplementary Materials

The following supporting information can be downloaded at https://www.mdpi.com/article/10.3390/ijms27135686/s1.

## Abbreviations

The following abbreviations are used in this manuscript:

Ab	Antibody(ies)
ALV-J	Avian leukosis virus subgroup J
BACΔMEQ	Bacterial artificial chromosome-derived MEQ-deleted Marek’s disease virus type 1 mutant
BoHV-1	Bovine herpesvirus type 1
BVDV	Bovine viral diarrhea virus
CIV	Canine influenza virus
DEV	Duck enteritis virus
DTMUV	Duck Tembusu virus
DVE	Duck viral enteritis
E	Envelope protein (used in WNV prM/E)
E2	Bovine viral diarrhea virus envelope glycoprotein E2
EHV-1	Equine herpesvirus type 1
EIV	Equine influenza virus
Env	Envelope protein (used in ALV-J)
Erns	BVDV envelope glycoprotein Erns (ribonuclease)
FMDV	Foot-and-mouth disease virus
g (gB, gJ, gIII)	Glycoprotein (e.g., glycoprotein B, J, or III of the respective herpesvirus)
GM-CSF	Granulocyte-macrophage colony-stimulating factor
GP5	Glycoprotein 5 (PRRSV)
HA	Hemagglutinin
HPAIV	Highly pathogenic avian influenza virus
HVT	Herpesvirus of turkeys (as in rHVT-LT)
IBDV	Infectious bursal disease virus
IBR	Infectious bovine rhinotracheitis
IBV	Infectious bronchitis virus
ILT	Infectious laryngotracheitis
ILTV	Infectious laryngotracheitis virus
LPAIV	Low pathogenic avian influenza virus
MDV	Marek’s disease virus (MDV-1 = serotype 1)
N	Nucleocapsid protein (IBV)
NA	Neuraminidase
PFU	Plaque-forming units
prM	Pre-membrane protein (WNV)
PRRSV	Porcine reproductive and respiratory syndrome virus
PRV	Pseudorabies virus
QMV	Specific BoHV-1 mutant strain used in the study
RABV G	Rabies virus glycoprotein
rHVT-LT	Recombinant herpesvirus of turkeys expressing ILTV antigens
S/S1	Spike protein/S1 subunit of spike protein (IBV)
VNA(s)	Virus-neutralizing antibody(ies)
VP1, VP2	Viral structural proteins (FMDV VP1, IBDV VP2)
vv	Very virulent (as in vv + MDV challenge strain)
WNV	West Nile virus
