# Supplementary material for: Recombinant Alphaherpesvirus Vectors in Veterinary Vaccinology: Platforms, Applications, and Translational Challenges
Source: Int J Mol Sci. 2026 Jun 24;27(13):5686. doi: 10.3390/ijms27135686 (PMC13362248; doi:10.3390/ijms27135686)
Supplement: Supplementary file 1 [file ijms-27-05686-s001.zip › ijms-4331356-supplementary.pdf]

Supplementary Methods S1: Detailed Literature Search Strategy and Quality Appraisal Framework.

S1.1. Complete Database-Specific Search Strings

Search date: 1 June 2026

1. PubMed (search fields: Title/Abstract):

("Bovine herpesvirus 1" OR "BoHV-1" OR "BHV-1" OR "Pseudorabies virus" OR "PRV" OR "Suid herpesvirus 1" OR "Marek's disease virus" OR "MDV" OR "Gallid alphaherpesvirus 2" OR "Equine herpesvirus 1" OR "EHV-1" OR "Duck enteritis virus" OR "DEV" OR "Mardivirus anatidalpha1" OR "turkey herpesvirus" OR "HVT") AND ("recombinant vaccine" OR "viral vector" OR "vectored vaccine" OR "DIVA" OR "challenge study" OR "herpesvirus vector" OR "foreign gene expression" OR "multivalent vaccine")
Filters: Publication date: 1990/01/01 to 2026/06/01; Language: English; Article types: Journal Article, Review.

2. Web of Science (search field: Topic):

TS=((“Bovine herpesvirus 1” OR “BoHV-1” OR “BHV-1” OR “Pseudorabies virus” OR “PRV” OR “Suid herpesvirus 1” OR “Marek's disease virus” OR “MDV” OR “Gallid alphaherpesvirus 2” OR “Equine herpesvirus 1” OR “EHV-1” OR “Duck enteritis virus” OR “DEV” OR “Mardivirus anatidalpha1” OR “turkey herpesvirus” OR “HVT”) AND (“recombinant vaccine” OR “viral vector” OR “vectored vaccine” OR “DIVA” OR “challenge study” OR “herpesvirus vector” OR “foreign gene expression” OR “multivalent vaccine”))
Filters: Years: 1990–2026; Document Types: Articles, Review Articles; Language: English.

3. Google Scholar (advanced search):

Due to the 256-character limit for basic queries, a simplified query was used: ("BoHV-1" OR "PRV" OR "MDV" OR "EHV-1" OR "DEV") AND ("recombinant vaccine" OR "viral vector") with the date range set to 1990–2026. The first 500 results for each vector platform (sorted by relevance) were screened, as Google Scholar does not support exhaustive Boolean retrieval of all records. Additional records were identified through citation chaining (snowballing) from the reference lists of included reviews and key original articles.

S1.2. Screening and Selection Workflow

- Records identified through database searching (after duplicate removal): $n = 1{,}247$
- Records screened (title/abstract): $n = 1{,}247$
  - 20% screened independently by two reviewers (A.M., S.G.F.) → inter-rater agreement: 94%; disagreements resolved by discussion.
  - 80% screened by a single reviewer (A.M.).
- Records excluded based on title/abstract: $n = 1{,}065$
  (Reasons: irrelevant vector platform, no vaccine evaluation, non-peer-reviewed format, non-English language, purely diagnostic/epidemiological studies without vaccine components)
- Full-text articles assessed for eligibility: $n = 182$

- Full-text articles excluded: $n = 61$
  (Reasons: insufficient efficacy data, lack of heterologous antigen expression, studies in non-target species without validation, duplicate data from same research group)
- Articles included in narrative synthesis (original research): $n = 82$
- Reviews/meta-analyses included for background: $n = 39$
- Additional records identified via reference chaining: $n = 23$
- Total included references: $n = 121$ (original research) + 39 (reviews) = 144 unique references cited in total, with 82 primary efficacy studies feeding into Table 2.

S1.3. Quality Appraisal Checklist for Included Original Research Articles

We assessed the 82 original research articles against the following criteria, adapted from the ARRIVE 2.0 guidelines and SYRCLE's Risk of Bias tool. Each criterion was scored as Yes (clearly reported), No (not reported or inadequately reported), or Unclear (insufficient information).

Criterion	Description
1. Sample size justification	Was an a priori sample size calculation or power analysis provided to justify the number of animals per group?
2. Randomization	Was random allocation to treatment groups described? Was the method of randomization specified (e.g., simple, block, stratified)?
3. Allocation concealment	Were the group assignments concealed from investigators until after allocation?
4. Blinding (performance bias)	Were the animals or caretakers blinded to the treatment allocation during the experiment?
5. Blinding (detection bias)	Were the outcome assessors blinded to the treatment groups during clinical scoring, histopathology, or virological assays?
6. Comparator groups	Were appropriate control groups included (e.g., unvaccinated, mock-vaccinated, or vector-only controls) from the same source population?
7. Challenge model validity	Was the challenge virus strain and route clinically relevant to natural infection? Was the challenge dose specified and justified?
8. Outcome measures	Were clinically relevant endpoints used (e.g., survival, clinical signs, viral shedding, pathological lesions)?

Criterion	Description
9. Statistical reporting	Were statistical methods clearly described? Were effect sizes and confidence intervals reported in addition to *p*-values?
10. Conflict of interest / funding	Were sources of funding and potential conflicts of interest declared?

Summary of quality appraisal findings:

Based on our assessment of the 82 original research articles:

Criterion	Proportion of studies meeting the criterion (%)
Sample size justification	18%
Randomization method specified	45%
Allocation concealment described	12%
Blinding (performance)	<10%
Blinding (detection)	28%
Appropriate comparator groups	95%
Valid challenge model	92%
Clinically relevant outcomes	100%
Effect sizes/confidence intervals reported	24%
Conflict of interest declared	78%

Interpretation: The primary methodological shortcomings across the reviewed studies are the lack of a priori sample size calculations, insufficient reporting of randomisation and blinding procedures, and limited presentation of effect sizes with confidence intervals. These issues are common in preclinical vaccine research and may lead to an overestimation of efficacy (publication bias) and reduced reproducibility. We have therefore reported protection estimates in Table 2 with appropriate caution and encourage future investigators to adhere to ARRIVE guidelines to improve evidence quality.
